# Field evaluation of a volatile pyrethroid spatial repellent and etofenprox treated clothing for outdoor protection against forest malaria vectors in Cambodia

**DOI:** 10.1038/s41598-024-67470-3

**Published:** 2024-07-29

**Authors:** Élodie A. Vajda, Amanda Ross, Dyna Doum, Emma L. Fairbanks, Nakul Chitnis, Jeffrey Hii, Sarah J. Moore, Jason H. Richardson, Michael Macdonald, Siv Sovannaroth, Pen Kimheng, David J. McIver, Allison Tatarsky, Neil F. Lobo

**Affiliations:** 1 University of California, San Francisco, 550 16th Street, San Francisco, CA 94158 USA; 2 Swiss Tropical and Public Health Institute, Socinstrasse 57, 4002 Basel, Switzerland; 3 University of Basel, Petersplatz 1, 2003 Basel, Switzerland; 4 Health Forefront Organization, Phnom Penh, Cambodia; 5 Vector Control Product Testing Unit, Environmental Health and Ecological Science Department, Ifakara Health Institute, P. O. Box 74, Bagamoyo, Tanzania; 6 Innovative Vector Control Consortium, Liverpool School of Tropical Medicine, Pembroke Place, Liverpool, Merseyside L3 5QA UK; 7 National Center for Parasitology, Entomology and Malaria Control, 477, Phnom Penh, Cambodia; 8 Department of Health of Mondulkiri, C5XX+CP4, 76, Krong Saen Monourom, Cambodia; 9 University of Notre Dame, Notre Dame, IN 46556 USA

**Keywords:** Behavioural ecology, Infectious diseases, Mathematics and computing, Computational science, Scientific data, Statistics

## Abstract

Cambodia’s goal to eliminate malaria by 2025 is challenged by persistent transmission in forest and forest fringe areas, where people are exposed to *Anopheles* mosquito bites during the day and night. Volatile pyrethroid spatial repellents (VPSRs) and insecticide-treated clothing (ITC) could address these gaps. This study evaluated the outdoor application of one passive transfluthrin-based VPSR, four etofenprox-ITCs paired with a picaridin topical repellent, and a combination of VPSR and ITC against wild *Anopheles* landing in Cambodia. A 7 × 7 Latin-square study was conducted over 49 collection nights in temporary open structures in Mondulkiri Province. All interventions substantially reduced *Anopheles* landing, with protective efficacy ranging from 61 to 95%. Mathematical modeling showed significant reductions in vectoral capacity, especially with the combined ITC and VPSR and VPSR alone, albeit with decreased effectiveness over time. These interventions have the potential to reduce outdoor and daytime *Anopheles* biting, offering valuable contributions to malaria elimination efforts in Cambodia and the Greater Mekong Subregion, contingent upon achieving effective coverage and adherence.

## Introduction

The Greater Mekong Subregion (GMS) aims to achieve elimination of *Plasmodium falciparum* malaria by 2025, and of all human malaria by 2030^1,2^. From 2000 to 2020, the GMS recorded a 56% decrease in malaria, and an 89% reduction in *P. falciparum* cases. As of 2022, Cambodia accounts for 2% of all malaria cases and for 1% of *P. falciparum* cases in the GMS. Operating on an accelerated timeline compared to the remaining GMS countries, Cambodia strives to eliminate *P. falciparum* malaria by 2024, and all human malaria by 2025^1^.

From 2010 to 2022, confirmed malaria cases in Cambodia declined by 91%. As malaria decreases throughout the country, it has become increasingly confined to malaria transmission foci^2^. Roughly 0.5 million people in Cambodia live in forest and forest fringe areas characterized by high malaria transmission^3,4^. These forest transmission foci are predominantly inhabited by ethnic minorities, local populations, and rural mobile and migrant populations working in rubber plantations, mining, and agriculture^5–7^. Under the Malaria Elimination Action Framework (2021–2025), the Cambodia’s National Center for Parasitology, Entomology, and Malaria Control deploys forest packs, often containing insecticide-treated nets (ITNs), insecticide treated hammock nets, and topical repellents to mobile and migrant populations in areas at highest malaria risk^2,8,9^. Since November 2020, Cambodia has also adopted foci-based innovative strategies as part of its “last mile” strategy towards *P. falciparum* malaria elimination, including targeted drug administration and intermittent preventive treatment for forest-goers^9,10^.

Appropriate mosquito bite prevention interventions to reduce forest-going and -dwelling populations’ exposure to forest-based *Anopheles* are needed based on the spaces and times where, and when these individuals are exposed to *Anopheles*^4^. A recent study conducted in northern Cambodia found that while *Anopheles* densities strongly declined in villages during the dry season, densities remained relatively similar from the dry to the wet season in the forest^7^. Therefore, forest sites may serve as suitable refuges for *Anopheles* during the dry season, and consequently, also as a malaria parasite reservoir, since human activity in the forest is particularly extensive during the dry season^7^. In addition, forest-goers are exposed to vector bites during the day due to *Anopheles* exhibiting outdoor, daytime and early evening biting^11^; and during the night, due to low bed net use and open sleeping structures^7,12^. These characteristics limit the effectiveness of strategies that focus on traditional village- and homestead-centric vector control interventions (indoor residual spraying (IRS), ITNs)^6,13^.

Additional interventions that target mosquitoes and people outdoors must be available for use alongside IRS and ITNs. This study focuses on evaluating two novel, promising tools: volatile pyrethroid spatial repellents (VPSRs), and insecticide treated clothing (ITC). VPSRs function by preventing human-vector contact primarily through non-contact irritancy, non-contact excito-repellency (the combined effects of both irritation resulting from coming into direct contact with a treated area and the tendency to avoid treated areas due to their repellent properties), spatial repellency, landing inhibition, feeding inhibition, sublethal incapacitation, and pre-/post-prandial (before/after blood feeding) mortality^14^. ITCs primarily protect humans from mosquito bites through contact irritancy, contact excito-repellency, some short-range non-contact excito-repellency, feeding inhibition, and mortality^15–17^. Synthetic topical repellents like picaridin (and DEET) offer personal defense against mosquito bites through short-range mechanisms such as blocking olfactory attractants^18,19^, inducing non-contact irritancy, and/or direct contact contact irritancy^20,21^. Pairing insecticide-treated clothing (ITCs) with a topical repellent may amplify bite prevention^22^.

VPSRs have been extensively evaluated in Africa^23–29^ and increasingly in Southeast Asia^30–33^. ITCs, treated with pyrethroids, have been widely evaluated and show promise for their use against *Anopheles* biting amongst mobile populations and military/ranger personnel^34–37^. However, commonly used permethrin formulations have shown poor wash retention and low bite prevention levels on many lighter weight fabrics prompting the development of etofenprox clothing treatment products with superior wash retention and safety profiles^38,39^. The evidence that permethrin treated clothing provides protection against malaria is unclear^16,17,40–42^ and the WHO currently does not recommend its use for the prevention and reduction of malaria at the community level where malaria transmission is ongoing^43^.

The study presented here is a component of a multi-staged research program called ‘Project BITE’ (Bite Interruption towards Elimination) (2020–2023)). Project BITE applied a mixed-methods, phased approach to vector control product evaluation^44^. Prior to the field intervention evaluation, a series of preceding semi-field system (SFS) experiments were conducted in Thailand to measure the primary effects (landing prevention) and the secondary effects (sublethal incapacitation and mortality) of new transfluthrin- and metofluthrin-based VPSRs, and etofenprox-treated clothing, against pyrethroid-susceptible *Anopheles minimus* (an important vector of malaria in the GMS^45^) (manuscript under review). In addition to providing insights and estimates of intervention impact on landing inhibition, sublethal incapacitation, and mortality, the data were used to parameterize a mathematical model to predict the potential of the tools to provide community protection^46^. Vectoral capacity is a measure of the ability of the vector population to transmit a disease, defined as the total number of potentially infectious bites that would eventually arise from all the mosquitoes biting a single infectious human on a single day^47^. Interventions that reduce vectoral capacity have the potential to confer community-wide protection leading to reduced malaria burden^48^.

Building on the SFS studies, an entomological field intervention evaluation was undertaken to evaluate these VPSR and ITC interventions on wild *Anopheles* landing using human landing catches (HLCs) in a small-scale, controlled field setting in Cambodia. The study’s secondary objectives were to 1) validate the results from the preceding SFS evaluation by measuring intervention effectiveness against local, wild *Anopheles* landing, 2) measure the hourly human landing rates (HLR) in the control structures, towards describing the local *Anopheles’* evening and nighttime biting trends, 3) confirm the *Anopheles* species identification from a subset of the HLC-collected specimens, and to 4) predict the reduction of vectoral capacity of these interventions using mathematical modelling. The potential of these tools to provide protection beyond personal protection is investigated by combining data from both the field study in Cambodia and the preceding semi-field studies in Thailand.

## Methods

### Study site

**Figure 1:**
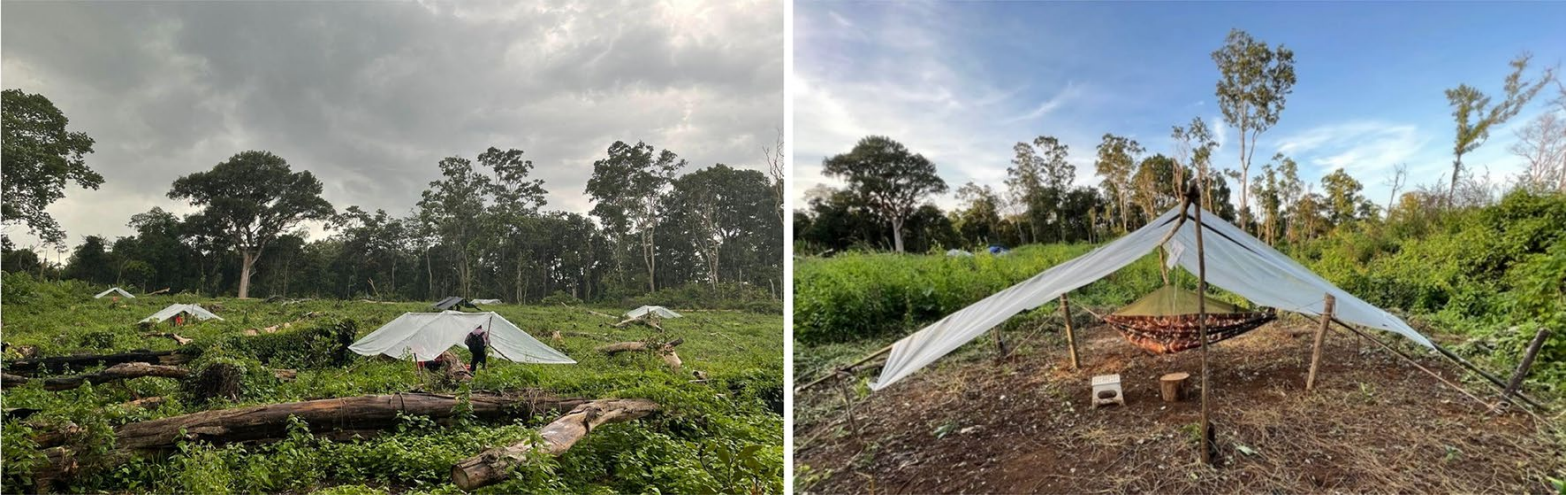
Temporary, open structures set-up in the field site in Andong Krolong, Mondulkiri Province, Cambodia (left), and front view of the temporary, open structures (right).

Field collections took place in the village of Andong Krolong (12.320725, 107.029779), Mondulkiri Province, Cambodia. The study took place from 23 September to 24 November 2022, thus taking place during the rainy season and the early days of the dry season. Temporary, open sleeping structures representative of open sleeping structures used by locals were constructed out of wooden poles and tarpaulin roofs (2 × 2 × 2 m) (Fig. 1). While closely surrounded by forest cover, the open structures were in an area that had been recently cleared, representative of the living conditions of forest workers (Fig. 1).

### Mosquito bite prevention interventions

Six bite prevention interventions were evaluated (Table 1). Interventions included a transfluthrin-based VPSR (BiteBarrier (‘BB’), formerly known as PIRK, PIC Corps), civilian and ranger clothing that were either newly treated with etofenprox or treated with etofenprox and then washed 20 times, as well as one arm with both the VPSR and etofenprox-treated civilian clothing. The treated civilian and ranger clothing was always paired with a 20% picaridin topical repellent (*SC Johnson*). The picaridin repellent was selected for this study as it has previously been demonstrated to be safe and effective against Southeast Asian vectors of malaria^11^.

**Table 1:** Descriptions of the mosquito bite prevention interventions and the control.

Intervention and control	Description
BB	A passive, transfluthrin-based VPSR (BiteBarrier, formerly known as PIRK, PIC Corps), composed of two 25 × 25 cm sheets: two units (four sheets total) of this product were hung about 1.5 m above ground, from opposing sides of the temporary, open structure. As the product manufacturer recommends replacing the product once every 30 days, the BB was replaced at the beginning of each seven-day collection rotation to ensure its efficacy did not wane during the experiments Additional product information: BB is transfluthrin-based (98.68%), an odorless active ingredient manufactured by *Bayer*, known for its excellent safety profile in mammals^49^. Depending on their exposure levels to this active ingredient, transfluthrin can effectively prevent mosquito biting and induce mosquito mortality^14,50^
EtoC-0-Pi	Etofenprox (Perimeter ETO Insect Guard formulation) -treated civilian clothing (unwashed) with a 20% picaridin (20 g/100 g) topical repellent (OFF! Tropical Strength Insect Repellent Spray, SC Johnson & Son Pty Ltd): 100% cotton short-sleeve t-shirt with ankle-length trousers. Clothing was treated by hand by the field team, using the etofenprox spray application bottles (700 mL spray bottles (7.0% etofenprox) with a calibrated nozzle. The etofenprox was applied according to manufacturer’s instructions: the bottle was held 15–20 cm away from the garment to allow spraying on fabric for a treatment level of 2.0 g/m^2^. Using slow, sweeping motion, the garments were evenly coated for approximately 30 s on each side. Garments were air dried for two hours by being hung outside Prior to each collection shift, the collector applied a 20% picaridin topical repellent (in a spritz-bottle) evenly to the forearms and hands. The application rate was 1 mL/600 cm^2^ Additional etofenprox product information: Etofenprox is an insecticide manufactured by *Mitsui Chemicals* and formulated exclusively by *Pine Belt Processing*, a wholly owned subsidiary of *Warmkraft, Inc*. Etofenprox is approved by the United States Environmental Protection Agency (US EPA) specifically designed for treating clothing worn by the US military^51^
EtoC-20-Pi	Etofenprox-treated civilian clothing (washed 20 times) with a 20% picaridin topical repellent: this is the same intervention as EtoC-0-Pi, except the clothes were hand washed 20 times with soap (mild soap similar to Savon de Marseilles) before use to simulate real-world use Additional washing information: For 1 kg of clothing, 20 g of mild soap was used for 8 L of water. Soap was dissolved in cold water (27 ± 3 °C). Clothes were soaked in soapy water for 10 min, then gently squeezed for 1–2 min and soaked again for another 2 min. This step was repeated twice. Clothing was then rinsed by gently squeezing clothing and soaking for 2 min in clean water. Clothes were then hung and allowed to dry in the shade
EtoR-0-Pi	Etofenprox-treated ranger uniform (unwashed), with a 20% picaridin topical repellent: etofenprox was applied to the ranger uniform and topical repellent was applied to the exposed hands, as for EtoC-0-Pi
EtoR-20-Pi	Etofenprox-treated ranger uniform (washed 20 times), with a 20% picaridin topical repellent: etofenprox was applied to the ranger uniform and topical repellent was applied to the exposed hands, as for EtoC-0-Pi
BB + EtoC-0-Pi	Two interventions were combined: VSPR and EtoC-0-Pi, both as above
Ctl	Negative control: collectors in the control arm wore 100% cotton, untreated, short-sleeve t-shirt and knee-length trousers, leaving the area between the knees and ankles exposed. A negative control with long trousers was not included, primarily due to resource and time constraints

### Experimental design and mosquito collections

Seven temporary, open structures located at least 20 m apart were set up. HLCs were carried out in the structures for 12 h, from 18h00 to 06h00, divided into two collection shifts, 18h00–00h00 (shift 1) and 00h00–06h00 (shift 2), where each shift was covered by a single collector per structure (for example, in structure 1, collector 1 worked shift 1, and collector 2 worked shift 2). A fully balanced 7 × 7 Latin-square design was used. Each of the seven study arms (six interventions and one control) was assigned to one structure for seven collection nights, and each pair of collectors rotated through each location on a nightly basis. After each block of seven collection nights, interventions were advanced to the next position, and collectors continued to rotate through structures each night. After 49 nights of collection each collector had tested each intervention in each location seven times. There were 20 unique HLC collector pairs. Some collector pairs worked fewer HLC nights than others as some individual collectors left the study before its completion. Due to cultural perceptions about being in the forest at night, collector pairs remained together in the structure throughout the entire collection night: while one collector worked, the other collector slept underneath the structure, in an untreated hammock net.

HLCs were used to collect mosquitoes landing on the area from knee to ankle of the collector for each collection rotation. For etofenprox-treated clothing interventions, long trousers were not rolled up to the knee in order to estimate the landing protection afforded by the combination of etofenprox with long trousers. The negative control had the area between the knee and the ankle exposed. The total number of mosquitoes caught hourly was recorded. Mosquitoes captured were stored in individually labelled Eppendorf tubes (by treatment and hour of collection), transported in coolers to the base camp every morning, killed by freezing, counted, morphologically sorted, and stored individually with desiccant in Eppendorf tubes for subsequent processing.

### Ethical statement

All methods were carried out in accordance with Cambodia’s National Center for Parasitology, Entomology, and Malaria Control guidelines; all HLC collectors were over 18 years of age, and provided written informed consent and were medically supervised, i.e., provided free-of-cost malaria diagnosis and treatment should malaria symptoms (e.g., fever) occur during the study and/or during the two weeks subsequent to the field trial period. Ethics approval for this study protocol was obtained from the Cambodia Ministry of Health’s National Ethics Committee for Health Research (nº296).

### Environmental data collection

Temperature (Celsius), % relative humidity, windspeed (m/s) and rainfall occurrence were recorded on an hourly basis during HLC collections, from 18h00 to 06h00. Temperature, % relative humidity, and wind speed data were recorded at the end of each HLC collection hour using a data logger device (HOBO^®^). Rainfall occurrence was recorded as a binary variable (yes/no) to indicate occurrence or absence of rainfall during each HLC collection hour.

### Mosquito morphological and molecular species identification

All *Anopheles* mosquitoes were sorted to species or species group using morphological identification keys^52^ in the field. Individual specimens were then packaged in individual, tightly closed, Eppendorf tubes with silica gel and were sent to University of Notre Dame, Indiana, USA, for molecular species confirmation. Approximately 15% of samples were randomly selected across all HLC collections, and sequenced at the ribosomal DNA internal transcribed spacer region 2 (ITS2) and/or cytochrome oxidase subunit 1 (CO1) loci towards species determination^12^. Conservative molecular species identification was based on matches to GenBank (National Center for Biotechnology Information [NCBI]) and BOLD^53^ (databases with lower quality matches and an absence of voucher specimens resulted in identifications to higher taxonomic levels).

### Data analysis

The change in landing associated with each intervention was estimated as rate ratios (RR), the ratio of the number of *Anopheles* landing in the intervention compared to that in the control. They were estimated using a mixed-effect negative binomial regression with structure ID, collector pair, and intervention ID as fixed-effects, and with collection date and batch (the location-night) as random effects. The effect of structure location and collector pair on *Anopheles* catches per collection night was also examined. All RR estimates are presented with 95% confidence intervals (CI). The percentage protective efficacies of each intervention against *Anopheles* landing were estimated as $\left(1-RR\right) \times 100$.

The relationship between weather variables and *Anopheles* densities caught was investigated. Mean nightly temperatures, mean nightly %RH, mean nightly wind speed, and number of rain occurrences per night were plotted against the total number of *Anopheles* captured per night. All statistical analyses were performed in R (Murray Hill, New Jersey), Version 2023.03.0 + 386^54^, using the tidyverse packages ‘tidyr’^55^, ‘dplyr’^56^, ‘lme4’^57^, and ‘ggplot2’^58^.

The hourly human landing rate (HLR) for the control arm across the 49 collection nights was estimated as a proxy for the hourly human biting rate^22,59^.

### Modeling of vectoral capacity

The relative reduction in vectoral capacity was predicted using the method described in Denz et al. (2021)^48^ and in Fairbanks et al.^46^. Firstly, the vectoral capacity is calculated for a “baseline scenario” (without the intervention). Parameters used in the calculation for this are given in Suppl Table 1. Biometric parameters for *Anopheles dirus* were used since this species accounted for most of the local mosquito population. Next, were considered other scenarios where a proportion of the population is protected by an intervention. This proportion is dependent on both the coverage (whether someone has the intervention) and adherence (whether someone uses the intervention).

Fairbanks et al.^46^ used data from semi-field studies combined with a model to quantify the modes of action of selected vector control tools. The influence of the interventions on the mosquito feeding cycle were described relative to an unprotected human host using four characteristics; the relative reduction in the rate of landing, the relative increase in the rate of preprandial killing and disarming, the proportion of this increase in rate which is due to disarming, and the change in the probability of postprandial mortality. Estimates from the field study are used to update some of these parameters to provide estimates for the wild *Anopheles* at this field site. For some interventions there are multiple semi-field estimates (from different sites and years), in this case we consider estimations from each semi-field scenario separately, and therefore have multiple estimates for the reduction in vectoral capacity for these interventions.

The relative reduction in the rate of biting parameters estimated from the field study were used. Then, to consider the increase in the rate of preprandial killing and disarming, the relative change in the rate of biting in the field studies compared to semi-field studies is first calculated:

$$\frac{{\uppi }_{\text{F}}}{{\uppi }_{\text{SF}}}$$

where ${\uppi }_{\text{F}}$ and ${\uppi }_{\text{SF}}$ are the median estimated protective efficacies for the change in the rate of biting in in the field and semi-field studies, respectively. This ratio helps to understand how the reduction in biting in the field studies compares to that in the semi-field studies. The reduction in the rate of disarming and preprandial killing is then calculated as:

$${\upkappa }_{\text{F}}= \frac{{\uppi }_{\text{F}}}{{\uppi }_{\text{SF}}} {\upkappa }_{\text{SF}}$$

where ${\upkappa }_{\text{SF}}$ is the estimated rate from the semi-field data. This scaling method ensures that the proportion of the reduction in biting attributed to disarming or preprandial killing matches that observed in the semi-field studies, while the magnitude of these effects are assumed to scale according to observation under field conditions (with wild *Anopheles*). The probability a mosquito is killed postprandially and the probability a mosquito is disarmed, given it is disarmed or killed preprandially, are assumed to be the same as in the semi-field conditions.

To incorporate variability and uncertainty, parameter values are sampled from the protective efficacy distribution (reduction in biting) or the from the semi-field study (all other parameters).

## Results

### *Anopheles* species identification and human landing rates

A total of 8,294 *Anopheles* specimens were collected with HLCs. Of this total, 96% (n = 7951) of specimens were morphologically identified to species or species group. Out of the total morphologically identified, 96% (n = 7621) were identified as *An. dirus* sl, leaving the remaining 5% of specimens identified to eight different species or species group: *Anopheles maculatus* sl (n = 234), *An. minimus* (n = 63), *Anopheles philippinensis* (n = 10), *Anopheles kochi* (n = 8), *Anopheles aconitus* (n = 7), *Anopheles barbirostris* sl (n = 5), *Anopheles baimaii* (n = 2), and *Anopheles asiaticus* (n = 1). Molecular speciation of 15% (n = 1242) specimens were evaluated, and confirmed that the morphological identification was extremely accurate (Table 2).

**Table 2:** *Anopheles* species and species group confirmed via molecular species identification.

		ITS2			CO1			
Molecular species or species (sub)group	Number of specimens	NCBI Coverage^1^ (%)	NCBI E-value^2^	NCBI % Identity^3^ (%)	NCBI Coverage^1^ (%)	NCBI E-value^2^	NCBI % Identity^3^ (%)	BOLD % ID^4^ (%)
*An. dirus* Form A*	449	99	0	99.87	99	0	98.51	100
Subgroup Leucosphyrus	444	98	0	93	99	0	98.51	100
An. maculatus*	202	95	0	100	99	0	98.95	100
An. minimus*	67	99	0	100	99	0	98.79	99.84
*An. dirus* sl	30	96	0	99.62	99	0	98.51	100
An.sawadwongporni*	18	99	0	99.30	94	0	100	100
An. kochi*	10	98	0	99.80	98	0	99.84	99.84
*An. rampae*	8	100	0	99.52	89	0	99.12	99.47
Group Annularis	7	98	0	99.78	96	0	92.44	99.68
An. dissidens*	2	100	0	99.27	99	0	99.38	100
An. aconitus*	1	100	2.00E-121	100	99	0	99.53	99.80
An. crawfordi*	1	98	0	99.21	97	0	99.60	99.66
An. jamesii*	1	–	–	–	99	0	99.36	100
An. pallidus*	1	99	0	100	96	0	92.44	99.68
Group Maculatus	1	99	0	95.95	99	0	98.95	100

^1^National Center for Biotechnology Information (NCBI) Coverage: the extent to which a specific group of organisms, genes, or sequences are represented in the databases and resources provided by the NCBI.

^2^NCBI Expected (E) value: the number of different alignments with scores equivalent to, or better than, expected to occur in a database search by chance. The smaller the E-value, the more significant the score and alignment.

^3^NCBI % Identity: the degree to which two nucleotide or amino acid sequences have matching residues at the same positions in an alignment^60^.

^4^Barcode of Life (BOLD) % ID: used to assess the similarity between the sequenced DNA barcode and the reference barcode. A higher % ID indicates a closer match between the two sequences and suggests a higher likelihood that the specimen belongs to the species represented by the reference BOLD barcode^53^.

*Voucher specimens.

**Figure 2:**
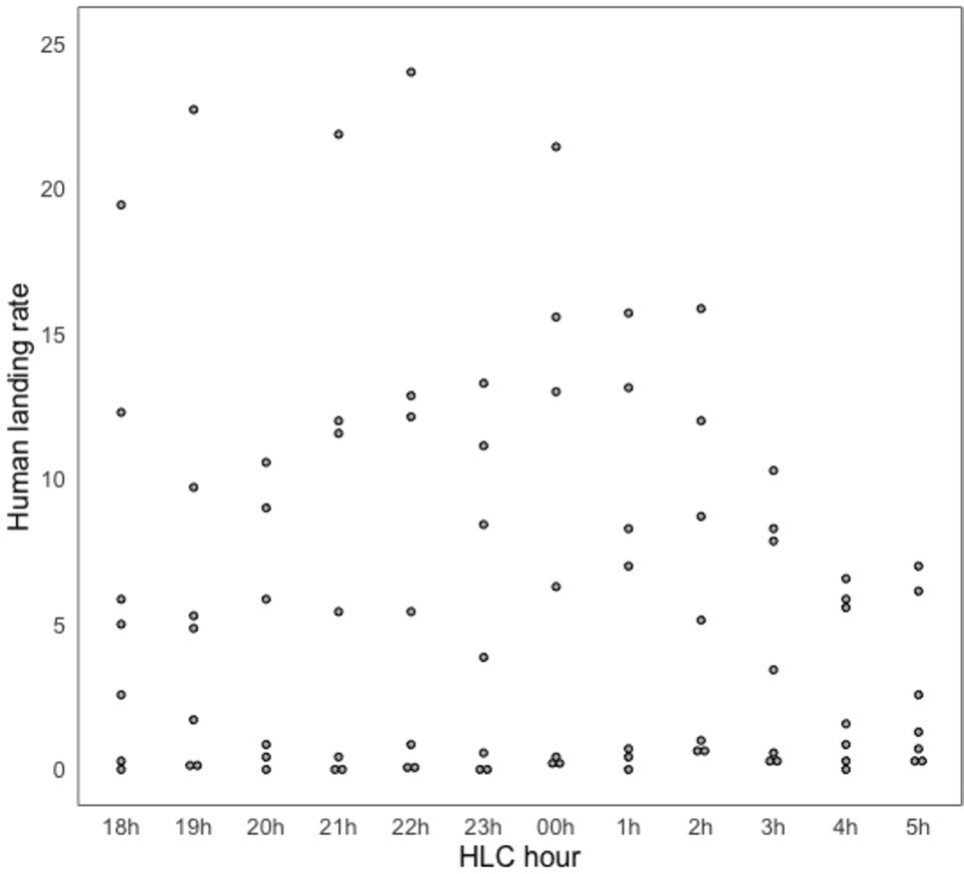
Hourly *Anopheles* human landing rate (HLR) (*Anopheles* landings per person, per hour), over the course of 49 collection nights (from control arms).

The estimated mean HLR per hour (control arms) ranged from 8.16 (95%CI 0.21–16.10) *Anopheles* landings per person per hour (lph) (00h00–01h00) to 2.61 (CI 0.00–5.22) lph (05h00–06h00). Higher landing rates were recorded from 18h00 to 00h00. After 02h00, a decline of the mean hourly HLR was observed until the last HLC collection hour (05h00–06h00) (Fig. 2).

### Intervention protective efficacy against *Anopheles* landing

**Figure 3:**
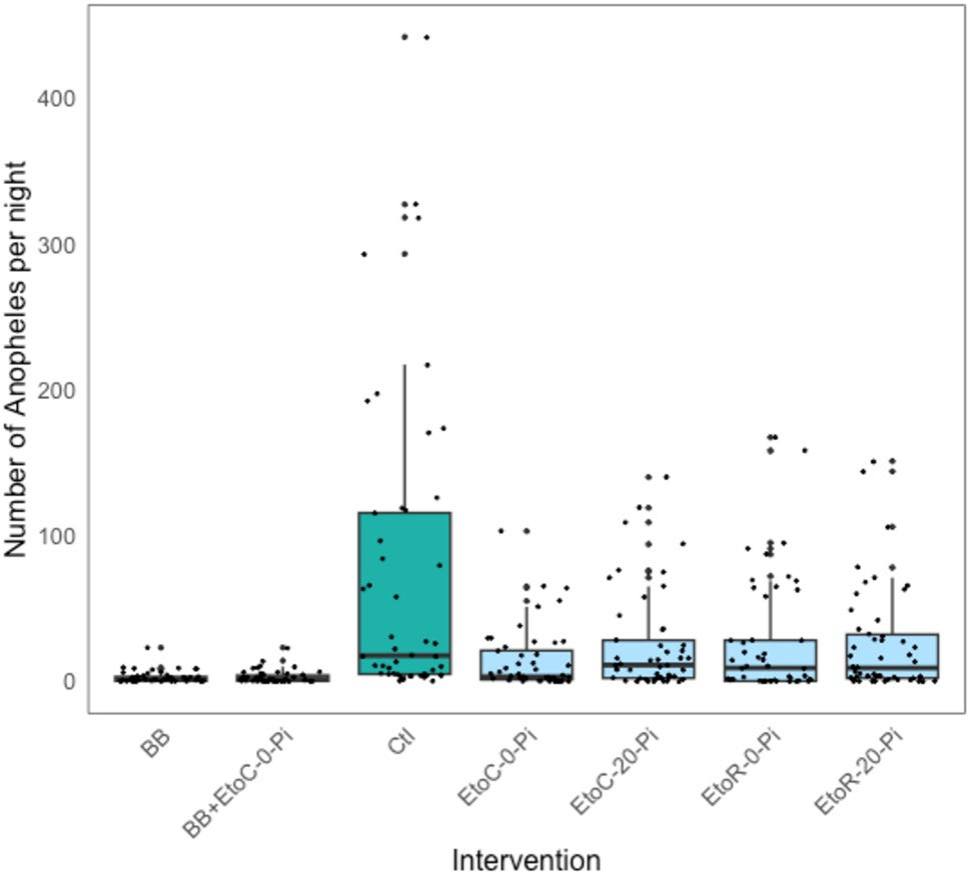
Number of *Anopheles* captured per HLC night by intervention.

**Figure 4:**
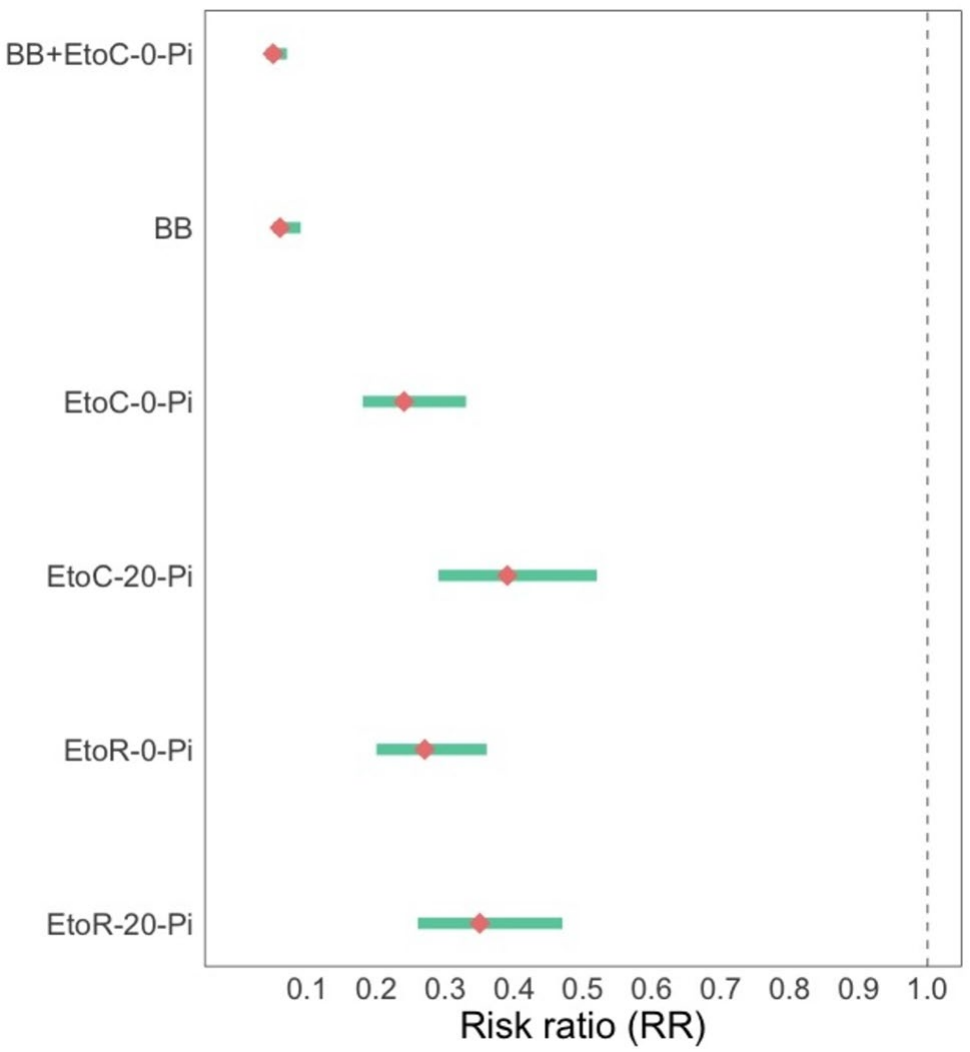
Estimated risk ratios (95% CI) for the effect of each intervention on risk of *Anopheles* landing (plotted on log scale).

All six mosquito bite prevention interventions showed substantial reductions in mosquito landings relative to the control (Ctl) (Fig. 3). The estimated protective efficacies of the interventions against *Anopheles* landing ranged from 61% (95%CI 48–71%) to 95% (CI 93–96%) (Fig. 4, Table 3). BB alone and the combined interventions (BB + EtoC- 0-Pi) provided the greatest protection against *Anopheles* landing, with protective efficacies of 94% (CI 91–95%) and 95% (CI 93–96%), respectively. The etofenprox-treated ranger uniform and civilian clothing, unwashed, provided similar protection against landing, 73% (CI 64–80%) and 76% (CI 67–82%), respectively. Both etofenprox-treated ranger uniform and civilian clothing continued to provide substantial protection after 20 washes, 65% (CI 53–74%) and 61% (CI 48–71%), respectively (Fig. 4, Table 3).

**Table 3:** Estimated rate ratios (RR) and their derived protective efficacies by intervention.

Intervention	RR	95% CI	*p*-value	Protective efficacy (%): 100 x (1-RR) (%)	PE 95% CI (%)
BB + EtoC-0-Pi	0.05	[0.04–0.07]	< 0.001	95	^93–96^
BB	0.06	[0.05–0.09]	< 0.001	94	^91–95^
EtoC-0-Pi	0.24	[0.18–0.33]	< 0.001	76	^67–82^
EtoC-20-Pi	0.39	[0.29–0.52]	< 0.001	61	^48–71^
EtoR-0-Pi	0.27	[0.20–0.36]	< 0.001	73	^64–80^
EtoR-20-Pi	0.35	[0.26–0.47]	< 0.001	65	^53–74^

Overall, there was variation in the number of *Anopheles* captured per night by both the structure locations (Suppl Fig. 1), and collector pair (Suppl Fig. 2), justifying the inclusion of location and volunteer pair as covariates in the statistical model. Environmental parameters (temperature, %RH, wind speed and rainfall) were examined against total *Anopheles* captures per night. Temperatures remained stable, between 22 and 24 °C, and wind speed did not exceed 1.2 m/s; there was no clear relationship between temperatures or wind speed with *Anopheles* captures. Humidity was highest (100%) during the period of heaviest rainfalls (October), which coincided with lower *Anopheles* catches.

### Relative reduction in vectoral capacity

This parameter was measured by combining field data and the semi-field data generated during the preceding SFS trials in Thailand to predict the potential of the tools to provide community protection.

**Figure 5:**
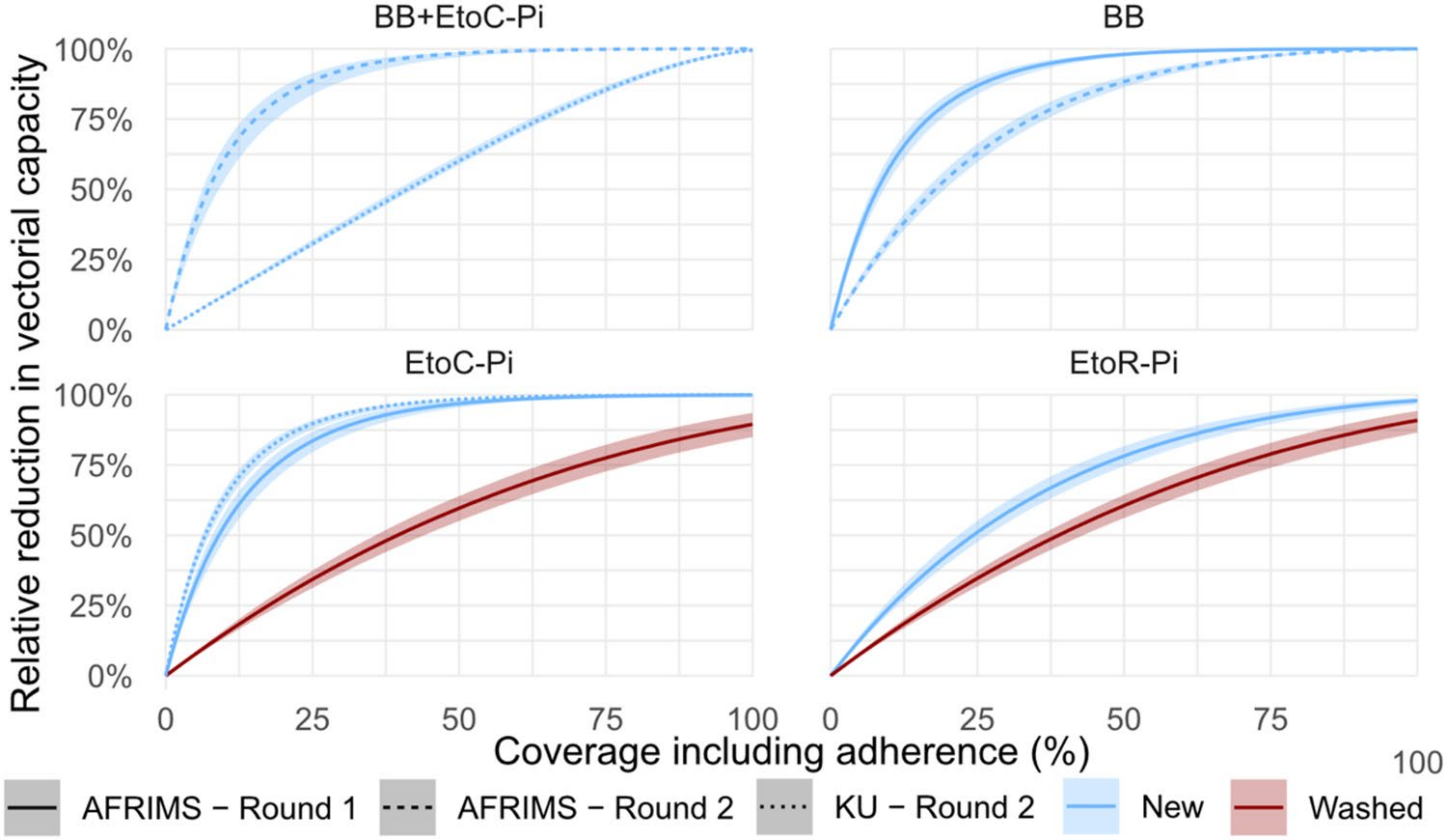
Predicted relative reduction in vectoral capacity for *Anopheles dirus* due to each intervention for a range of coverage levels for each intervention trailed in the field experiments. For interventions with semi-field estimates from multiple sites and years an individual line is plotted for each semi-field scenario. Black lines represent the median estimates, and colored bands represent the 95% CIs.

The relative reduction in vectoral capacity for each intervention coverage is shown in Fig. 5. Overall, all interventions were predicted to have a large impact on the vectoral capacity. The impact increases with coverage and adherence. In line with the reduction in landing observed in the field study, the greatest reduction of vectoral capacity was predicted for the combined EtoC and BB VPSR as well as the BB VPSR used alone. Even at 50% usage large reductions in vectoral capacity are predicted. As interventions age, and consequently dosage of available insecticide deceases, they are less effective at reducing the vectoral capacity (Fig. 5).

Although predictions have similar trends when utilizing data from different semi-field experiments, the magnitude of the predicted reduction in vectoral capacity is variable. There were differences in the extent of the modes of action observed, and therefore the parameter estimates, between experiments. These could be due to a number of factors including meteorological differences between locations and times and genetic differences between mosquito colonies.

## Discussion

In this field evaluation, the authors measured the impact of outdoor use of a passive, transfluthrin-based spatial repellent and etofenprox-treated clothing (paired with a 20% picaridin topical repellent) in the form of six bite prevention interventions on wild *Anopheles* landing. The study was designed to estimate landing rates (as a proxy for biting rates) when using the interventions while sitting in a fixed position in open structures similar to the open shelters used by forest-exposed populations in Cambodia.

A wide diversity of *Anopheles* species was observed in the study site in Mondulkiri (Table 2). Of the 15 *Anopheles* species collected, five are known vectors of human malaria: *An. dirus* Form A^12,61,62^, *An. maculatus*^63,64^*, An. minimus*^64,65^, *An. sawadwongporni*^64^, and *An. aconitus*^7,66^. Species *An. dirus*, *An. maculatus*, and *An. minimus* comprise Cambodia’s major malaria vectors^5,67,68^, and all 15 collected species have been previously identified in Cambodia^7,12,64^. While HLC collections occurred from early evening (18h00) to early morning (06h00), it is possible that daytime biting would also be observed had collections taken place prior to 18h00. Two other entomological studies in Mondulkiri province, one being in a forested site^4^, and the other being in both forested and village sites^7^, included 24-h *Anopheles* collections demonstrating daytime biting. Daytime biting suggests a further need for additional bite prevention interventions that are protective even when users are awake and outdoors^4,7^.

This field study found that all of the study interventions, even when washed (for the treated clothing interventions), provided substantial protection against wild *Anopheles* landing outdoors in open temporary structures (Table 3, Figs. 3, 4). The BB was highly effective against *Anopheles* landing, preventing 94% (CI 91–95%) of landings (Table 2). Combining this passive VPSR with etofenprox-treated clothing made no substantial difference on the level of protection provided against *Anopheles* landing. However, the combined intervention—delivered as a forest pack—is intended to protect real-world users both while inside their homes or temporary shelters and also while mobile (outside their homes/shelters), offering “full-time” or 24-h protection. In other words, the combined interventions provide more complete protection as needed based on individuals’ daily activities. However, for this study, the HLC collectors were restricted to the temporary structures.

Etofenprox-treated clothing interventions also provided high levels of protection against mosquito landing, though with slightly lower estimated protective efficacies than the BB intervention. However, while landing rates are often used as a proxy for measuring biting rates^22^, evidence indicates that landing might not always necessarily lead to biting. When mosquitoes are exposed to pyrethroids (airborne or applied to clothing), they may still be able to detect the host and land, but they are inhibited from biting^69^. In a lab study, the landing and biting rates of *Aedes aegypti* were measured while using metofluthrin VPSRs, and observed 74 landings for only eight bites^70^. Therefore, it cannot be excluded that measuring landing rates may lead to an overestimation of ‘biting’ and an under-estimation of protection. To more accurately estimate the protective efficacy of ITCs, it might be best to allow mosquitoes to feed as a more definitive endpoint of protective efficacy^71^.

This study only evaluated etofenprox-treated clothing paired with a topical repellent, as the preceding SFS trials demonstrated that the combined ITC and topical repellent intervention was more effective against mosquito landing than the treated clothing alone (Vajda et al.^37^, unpublished 2020/2021 data (manuscript under review)). In another field study evaluating personal repellent and treated clothing interventions in Lao PDR, permethrin-treated short clothing paired with a topical repellent were found to provide substantially more protection against mosquito landing than the permethrin-treated short clothing alone^22^. However, the permethrin-treated *long* clothing provided much higher protection against biting than the treated *short* clothing, but treated long clothing with a topical repellent was not tested^22^. Given the challenges with adherence around topical repellents use^17,20,72^, it would be useful to compare treated long clothing alone, to treated long clothing with a topical repellent. This would help better understand how much more protection against biting is conferred by the addition of a topical repellent.

On the other hand, this study included a negative control with *short* trousers, but not one with *long* trousers (due to resource and time constraints). However, because the etofenprox-treated clothing interventions comprised long trousers that were *not* rolled up to the knees during HLCs, it is possible that the ratios of *Anopheles* landing in the treated clothing interventions compared to the *Anopheles* landing in the control slightly overestimate the landing protective efficacy since the negative control did not have the added physical barrier provided by ankle-length trousers^73^.

Washing the etofenprox-treated clothing interventions 20 times only slightly increased the risk of *Anopheles* landing. This finding has implications for determining the frequency of retreatment of etofenprox-treated clothing. Efficacy testing of ITCs in controlled field and/or laboratory settings should be regarded as a proxy for real-world field conditions, in which ITCs would face harsher and more variable conditions (e.g., intense sweating during physical labor, textile degradation). Therefore, care should be taken when interpreting controlled testing results, and where possible, test results should be considered alongside results from efficacy testing in real-world field settings as is conducted for other vector control tools such as ITNs^74^.

Permethrin is the most common insecticide used to treat clothing^75^. Given the shortcomings of permethrin regarding longevity, efficacy on lightweight fabrics, and higher human toxicity (g/kg), etofenprox formulations for clothing treatment have been developed and registered by the U.S. Environmental Protection Agency^76^. Etofenprox is a synthetic pyrethroid-like ether insecticide. The structure of etofenprox renders it more stable and with lower mammalian toxicity than other pyrethroid insecticides and functions by attacking the neuronal axon of the mosquito^77^. As concerns over the spread of pyrethroid resistance grows, and several studies of the impact of pyrethroid resistance on the protective effect treated clothing yield conflicting results^38,39,78,79^ there is growing need to explore alternative insecticides for clothing treatment.

The set replacement interval of the BB device in the field must also be based on its residual efficacy. This study only tested new, freshly manufactured BB products towards providing a baseline understanding of the product’s efficacy against local, wild *Anopheles,* and did not investigate its residual efficacy. Therefore, the BB’s residual efficacy is currently unknown. Similarly to ITCs, transfluthrin-based VPSR efficacy over time is dependent on exposure to UV and other environmental parameters (rainfall, wind), as well as the susceptibility of local mosquitoes to pyrethroids^23,24,59,59,70,80^. For this field study, insecticide susceptibility testing to pyrethroids, including etofenprox and transfluthrin, was not feasible due to issues with keeping field-caught adult *Anopheles* alive and other logistical challenges.

This intervention evaluation provides evidence on the efficacy of these bite prevention interventions under controlled field conditions against wild *Anopheles* landing in Mondulkiri Province, Cambodia and highlights their potential for use in this elimination scenario if effective coverage and adherence of at-risk populations can be achieved. As with all vector control interventions, effectiveness is dependent on local vector bionomics^81^, vector insecticide susceptibility profiles and resistance mechanisms^82–84^, which uniquely affect intervention functionality^19,85–91^. Ecological, and human behavioral factors are also essential components that impact intervention effectiveness^51,92^. Therefore, malaria control and elimination programs must tailor their strategies and mix of interventions based on generated country-specific evidence and unique circumstances.

To date, the WHO has not established a position statement regarding the applications of VPSRs, ITCs, and topical repellents in public health vector control. However, while refraining from establishing formal recommendations on ITCs and topical repellents applications, the WHO does suggest these interventions for personal protection and considers their use for high-risk groups who do not benefit from other vector control interventions^43,93^. Recent work on a stochastic transmission model based on time-stratified vector landing data from controlled experiments of transfluthrin-treated eave ribbons (a type of VPSR) found that in addition to *Anopheles* vector landing reduction (personal protection), transfluthrin-treated ribbons also killed and reduced blood feeding, causing important reductions in vectoral capacity, indicating its potential to offer community protection^48^. The modeling evaluation conducted for this field study’s interventions’ on vectoral capacity also indicates reductions in vectoral capacity, even at decreased intervention coverage and adherence (Fig. 5). Limitations for the vectoral capacity model are described in detail in the original publication^94^. Here, parameters describing the bionomics of vectors in the model simulations were derived from averages for *An. dirus* from the GMS and surrounding areas^95^. However, these parameters, as well as different species of malaria vectors, can vary across different settings, potentially affecting the generalizability of our results. This model also assumed uniform changes in biting reduction and other endpoints between the SFS and field environments, based on data collected specifically on biting reduction. However, it is possible that other endpoints, such as mortality rates or incapacitation, may exhibit different patterns of change in field conditions compared to those observed in SFS. Still, this modeling assessment corroborates findings from recent SFS experiments^23–27,29^ and field studies of VPSRs^30,96,97^. Given this growing body of evidence, insecticidal personal protection interventions are increasingly recognized as having high potential for public health use, but further evidence of epidemiological impact is needed for WHO to establish a policy recommendation for these interventions^98^.

## Conclusion

In Southeast Asia, *Anopheles* bites occurring outside the protection of the traditional, homestead-centric interventions (IRS, LLINs), constitute important gaps in protection that call for novel bite prevention interventions. This field study is highly encouraging as it demonstrates that this transfluthrin-based BB VPSR and etofenprox ITCs provide substantial protection against *Anopheles* landing when used outside. Further, the study’s modeling analysis predicted that these interventions would reduce vectoral capacity, even at 50% coverage. Additional studies in other geographic settings are needed to estimate intervention impact on landing of mosquitoes with different bionomics profiles, and effectiveness trials in which the products are distributed and used normally are also required. While further evidence of epidemiological impact is necessary for WHO to establish these tools as effective public health interventions against malaria, this study provides promising results for bite prevention tools that can be used toward addressing gaps in protection of at-risk groups against *Anopheles* biting. In fact, recently, the U.S. President's Malaria Initiative (PMI) updated its technical guidance, enabling program evaluation of new tools, including spatial repellents, influenced in part by the positive outcomes from Project BITE and other recent and ongoing studies, reflecting an adaptive donor environment.

### Supplementary Information

Supplementary Information 1.

Supplementary Information 2.

Supplementary Information 3.

Supplementary Information 4.

## Data Availability

All data generated or analysed during this study are included in this published article and its supplementary information files.
