# Supplementary material for: Field evaluation of a volatile pyrethroid spatial repellent and etofenprox treated clothing for outdoor protection against forest malaria vectors in Cambodia
Source: Sci Rep. 2024 Jul 29;14:17348. doi: 10.1038/s41598-024-67470-3 (PMC11284218; doi:10.1038/s41598-024-67470-3)
Supplement: Supplementary file 4 — Supplementary Information 4. [file 41598_2024_67470_MOESM4_ESM.docx]

**Supplementary materials**

**Suppl Table 1** Definitions and values of parameters used to calculate the vectoral capacity. Baseline values, considering all humans as unprotected, for *Plasmodium falciparum* malaria and *Anopheles dirus* are given.

| Definition | Value | Ref. |
| --- | --- | --- |
| Number of humans | 10,000 | Assumption |
| Number of non-human hosts | 10,000 | Assumption |
| Maximum time a mosquito unsuccessfully searches for a blood meal per day | 0.33 days | Briët et al. 2019 [99] |
| Probability that a mosquito bites after encountering a host | 0.95 | Briët et al. 2019 |
| Probability that a mosquito finds a resting place | 0.95 | Briët et al. 2019 |
| Probability that a mosquito survives the resting phase | 0.99 | Briët et al. 2019 |
| Probability that a mosquito lays eggs and returns to host-seeking | 0.88 | Briët et al. 2019 |
| Time between feeding and laying eggs | 3 days | Wang et.al. 2024 [95] |
| Duration of the extrinsic incubation period (time required for sporozoites to develop in the mosquito) | 10 days | Briët et al. 2019 |
| The sac rate of mosquitoes (estimated proportion of host-seeking mosquitoes which laid eggs the same day) | 0.33 | Wang et.al. 2024 |
| The parity rate of mosquitoes (proportion of host-seeking mosquitoes that have previously laid eggs) | 0.64 | Wang et.al. 2024 |
| Human blood index (proportion of blood-fed mosquitoes which fed on a human) | 0.87 | Wang et.al. 2024 |
| Human availability rate (the rate at which mosquitoes encounter humans) | 8.51$\times{10}^{-5}$ $\mathrm{days}^{-1}$ | Calculated (as described in Briët et al. 2019) |
| Non-human availability rate (the rate at which mosquitoes encounter animals) | 1.27 $\times{10}^{-4}$ $\mathrm{days}^{-1}$ | Calculated (as described in Briët et al. 2019) |
| Per-capita mosquito death rate while searching for a blood meal | 0.22 $\mathrm{days}^{-1}$ | Calculated (as described in Briët et al. 2019) |

| **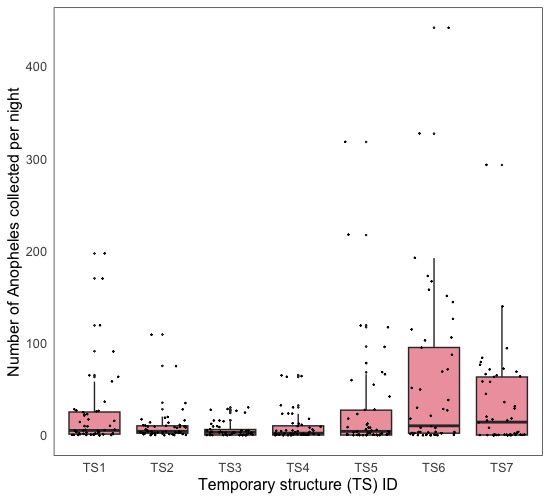**  **Suppl Fig. 1** *Anopheles* captured per HLC night, per temporary structure location. | **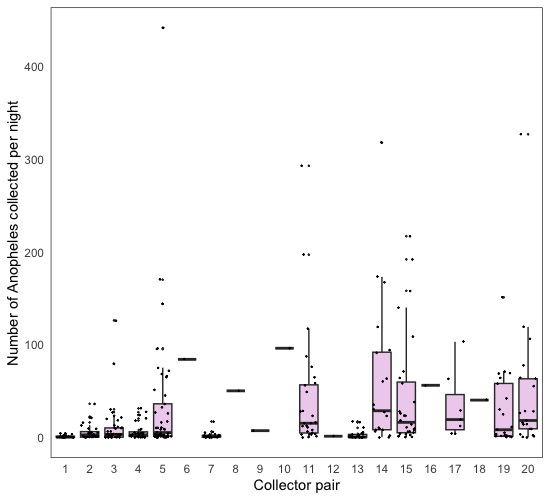**  **Suppl Fig. 2** *Anopheles* captured per HLC night, per collector pair. |
| --- | --- |
